# Cardiopulmonary Profiling of Athletes with Post-Exertional Malaise after COVID-19 Infection—A Single-Center Experience

**DOI:** 10.3390/jcm12134348

**Published:** 2023-06-28

**Authors:** Simon Wernhart, Eberhard Weihe, Matthias Totzeck, Bastian Balcer, Tienush Rassaf, Peter Luedike

**Affiliations:** 1 Department of Cardiology and Vascular Medicine, West German Heart- and Vascular Center, University Hospital Essen, University Duisburg-Essen, Hufelandstrasse 55, 45147 Essen, Germany; 2 Institute for Anatomy and Cell Biology, University Marburg, Robert-Kochstrasse 8, 35037 Marburg, Germany

**Keywords:** COVID-19, post-exertional malaise, 10-item DSQ-PEM

## Abstract

(1) Background: Cardiopulmonary exercise testing (CPET) has been suggested by the European Society of Cardiology (ESC) for assessing the exercise limitations of apparently healthy individuals, but data on elite athletes regarding this test are scarce. (2) Methods: We analyzed CPET in elite (n = 43, 21.9 ± 3.7 years) and recreational (*n* = 40, 34.7 ± 13.0 years) athletes with persistent subjective exercise intolerance and post-exertional malaise (PEM) after COVID-19 infection. The primary outcome was the point prevalence of the adequate cardiopulmonary response (ACPR), defined by the presence of all of the following ESC criteria for apparently healthy individuals: (1) >100% of predicted peak oxygen consumption (predVO2peak), (2) VE/VCO2 < 30, (3) no exercise oscillatory ventilation (EOV), and (4) heart rate recovery of ≥12 beats/minute 1 min after exercise termination (HRR1). Results: ACPR occurred more frequently in elite athletes than in recreational athletes (70.0% vs. 39.5%; *p* = 0.005), mainly driven by the lower VE/VCO2 (<30: 97.7% vs. 65%, *p* < 0.001). Elite (11.6%) and recreational athletes (22.5%) showing a plateau of O2 pulse did not display ACPR. Conclusions: ACPR was not observed in all recreational and elite athletes with PEM. In particular, perturbed VE/VCO2 and the plateauing of O2 pulse are suitable for quantifying exercise limitations and may identify a high-risk population with long-COVID-19 syndrome who require their training intensities to be adapted.

## 1. Introduction

Coronavirus-19 disease (COVID-19) has become a major challenge for physicians, not only in patient care [1] but also in treating and counseling professional and recreational athletes [2]. While there have been reports on severe short-term exercise limitations after COVID-19 infection, ranging from reduced peak oxygen consumption (VO2peak) and hyperventilation to reduced peripheral oxygen extraction [3,4,5], no data are available on the performance of recreational and elite athletes with persistent exercise intolerance. Although cardiorespiratory fitness expressed by VO2peak has been shown to be higher in non-hospitalized patients with COVID-19 infection as opposed to patients admitted to hospitals or even to the intensive care unit [6], hospitalization rates in athletes have been shown to be low [7]. However, a considerable decline in VO2peak has been demonstrated recently in endurance athletes following mild COVID-19 infection [8].

The post-acute sequelae of COVID-19 (PASC) syndrome subsumes more than 200 symptoms, including fatigue, dyspnea on exertion, headache, orthostatic intolerance, and post-exertional malaise (PEM) [9]. Although participation in everyday life can be severely compromised in these patients, a physiological definition of this complex is lacking. Limitations in cardiopulmonary exercise testing (CPET) have been suggested as being able to reveal endothelial damage as a (among others) potential correlate of PASC and PEM, and protocols for identifying these patients have been discovered [9,10]. 

PEM, which has been described as a manifestation of PASC [9], may be associated with persistent exercise intolerance as well as the development of long-COVID-19 syndrome [11]. PEM has been defined as an exacerbation of physical symptoms and a reduction in function after physical work, to an extent, which was not present before the illness [11,12,13]. PEM can be diagnosed with the validated 10-item De Paul Symptom Questionnaire (DSQ-PEM) [13]. The two-step score has been recommended by the National Institute of Neurological Disorders and Stroke Common Data Elements PEM Working Group [14].

There has also been increasing concern regarding the long-term cardiopulmonary complications of COVID-19 infections and the development of long-COVID-19 syndrome in athletes with PEM [15,16]. Better CPET characterization and quantification of exercise limitations in athletes with PEM are warranted to assess athletes’ risk of developing long-COVID-19 syndrome and to tailor adequate training intensities or even recommend refraining from exercise in cases of severely compromised athletes.

We followed the current threshold recommendations of the European Society of Cardiology (ESC) for CPET assessment in apparently healthy male and female individuals, defined as the presence of all of the following criteria to assess an adequate cardiopulmonary response (ACPR): (1) VO2peak ≥ 100% of predicted (predVO2peak), (2) VE/VCO2 < 30 (ventilatory class I), (3) no presence of exercise oscillatory ventilation (EOV), and (4) heart rate recovery of ≥12 beats/minute 1 min after exercise termination (HRR1) [17]. Usually, neither elite nor recreational athletes are expected to fall below these thresholds due to an expected higher fitness level compared to that of “normal” apparently healthy individuals. In the present study, we hypothesized that symptomatic elite and recreational athletes all display ACPR (the primary outcome). An analysis of the CPET variables in athletes may serve to reveal objective limitations of exercise performance to better characterize PEM and may help to better assess the risk of developing long-COVID-19 syndrome.

## 2. Materials and Methods

### 2.1. Setting and Participants

In this cross-sectional pilot study, we retrospectively analyzed elite and recreational athletes reporting to our outpatient clinic for a medical investigation due to persistent exercise intolerance and PEM as a potential residuum of COVID-19 infection [13]. Exercise intolerance was subjectively reported by the participants, and PEM was diagnosed using DSQ-PEM in all participants [13]. We used the validated 10-item DSQ-PEM consisting of two steps of evaluation to diagnose and assess the severity of PEM [13,14]. In the first scoring step, a threshold of 1 or more must be exceeded for the first five DSQ-PEM items. A score of 2–4 for frequency (half the time, most of the time, all of the time) together with a score of 2–4 for severity (moderate, severe, or very severe) for the same item was considered diagnostic for PEM. The supplementary second part contains questions on exercise exacerbation, quick recovery, and PEM duration. PEM requires an answer of “yes” for items 7 and 8, while a response of ≥14 h is required for item 9.

**Figure 1:**
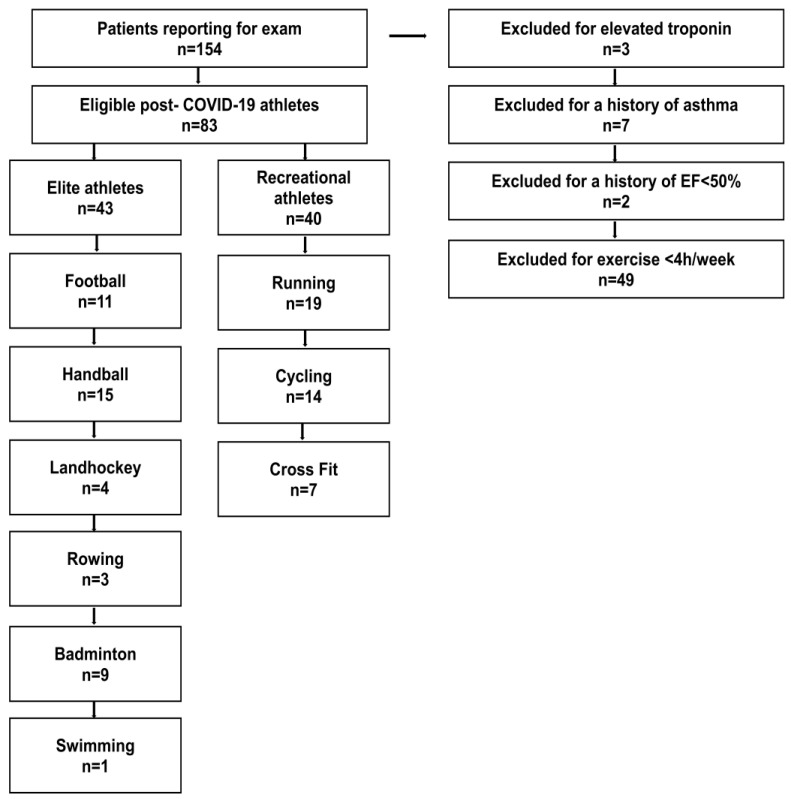
Workflow for study inclusion. EF: ejection fraction. COVID-19: coronavirus disease 2019.

The definition of elite and recreational athletes was made according to the current guidelines on sports cardiology [18,19]. Participants underwent a clinical investigation, laboratory diagnostics (including high-sensitivity troponin and NTproBNP), electrocardiography, and transthoracic echocardiography on the same day. The participants were instructed not to perform physical training within 48 h hours prior to troponin testing to reduce the risk of exercise-induced troponin elevation [15]. CPET was performed on the day of reporting to our outpatient clinic after verifying the absence of contraindications [15]. All participants had suffered from one documented SARS-CoV-2 infection, which had occurred >6 months before their inclusion in this study. None of the athletes had received treatment for COVID-19 and/or immunomodulant treatment. Disease severity was assessed according to the current guidance criteria of the World Health Organization [20]; only patients with a mild clinical course were included. Participants had to be >18 years of age without a medical history of cardiac, pulmonary, or muscular disease. All participants reported that they were willing to resume their participation in active high-intensity sports upon the clinical resolution of PEM and medical clearing. Abnormalities in the clinical exam, cardiac enzymes, electrocardiography, or echocardiogram led to further imaging (MRI and/or CT) and exclusion from the study (for a study workflow, see Figure 1). The study was approved by the local ethics committee (22-10586-BO).

### 2.2. Cardiopulmonary Exercise Protocol

Standard echocardiography was performed following the current guidelines [21]. CPET was performed on a bicycle ergometer (eBike II, GE Healthcare, Chicago, IL, USA) using standard software (SentrySuiteTM Software Solution, VyaireTM Medical, Hoechberg, Germany) with an exertional ramp protocol (incline of 30 W/min in each participant; pedaling rate of 60 RPM), defined as an RER > 1.05, with an estimated exercise duration between 8 and 12 min. Ventilatory thresholds were determined by an experienced sports scientist and sports cardiologist according to the current recommendations [17,22] using breath-by-breath gas exchange measurements with a metabolic cart interface (VyntusTM CPX Metabolic Cart, Vyaire Medical, Hoechberg, Germany).

The percentage of age-predicted VO2peak was determined using the Wasserman–Hansen equation [23]; exercise oscillatory ventilation (EOV) was calculated as initially described [24]. O2 pulse was expressed as related to body weight and multiplied by 100, as previously described [25]. To assess the increase in stroke volume during exercise and the extent of peripheral oxygen utilization, values of O2 pulse were determined at rest (O2 pulserest), 25% (O2 pulse25), 50% (O2 pulse50), and 75% (O2 pulse75) of exercise time, as well as at peak exercise (O2 pulsemax). A lack of increase in O2 pulse from one time point to the other was defined as the plateauing of O2 pulse. The cardiorespiratory optimal point, the oxygen equivalent at the first ventilatory threshold (EqO2 at VT1), was set at the nadir of EqO2 according to the current guidelines [17,26]. The oxygen uptake efficiency slope (OUES) was derived from the relation of logarithmic minute ventilation and oxygen uptake, as previously suggested [27]. Additionally, the OUES was also expressed as related to body surface area, as recently proposed [28]. The VE/VCO2 slope was determined from the exercise onset to peak exercise by linear regression [29]. The criteria for premature exercise termination were defined according to the current guidelines [17].

We defined an ACPR, according to the threshold values of current ESC guidelines in apparently healthy individuals [17], by the presence of all of the following criteria: (1) VO2peak ≥ 100% of predicted (predVO2peak), (2) VE/VCO2 < 30, (3) no presence of EOV, and (4) HRR1 of ≥12 beats/minute 1 min after exercise termination [17]. An inadequate cardiopulmonary response (ICPR) was defined as the presence of at least one of the following criteria: (1) <100% of predVO2peak, (2) VE/VCO2 > 30, (3) presence of EOV, and (4) HRR1 of <12 beats/minute. The primary outcome was the point prevalence of ACPR in elite and recreational athletes. 

We also analyzed standard maximal and submaximal CPET parameters as well as the impact of the ACPR and sports category (elite vs. recreational athletes) on the OUES and the difference between the resting and peak heart rate (ΔHR). Between-group differences for PEM symptoms were also analyzed.

### 2.3. Statistical Methods

Descriptive statistics and non-parametric tests were performed with SPSS (IBM Corp. Released 2016. IBM SPSS Statistics for Windows, Version 24.0. IBM Corp.: Armonk, NY, USA), while multivariable linear regression analysis was conducted with the R-program [30]. Normal distribution was tested using the Shapiro–Wilk test, nominal variables were evaluated using a chi-square test, and post hoc between-group comparisons were calculated using a nonparametric Mann–Whitney U-test. As a level of significance, α was set at 0.05. To estimate the impact of the sports category (elite vs. recreational athlete) and ACPR on the variables of VO2peak, OUES, and ΔHR, while accounting for age and sex as potential confounders, a multivariable linear regression model with a stepwise elimination of non-significant parameters was applied. Post hoc comparisons between categories and the cardiopulmonary response were calculated with an exact Wilcoxon–Mann–Whitney test. To determine a difference in the point prevalence of ACPR, we calculated a necessary sample size of *n* = 36 with a determination coefficient of R2 = 0.26, α = 0.05, and a power of 0.80 [31]. This is in the range of a recently published study comparing CPET data in endurance athletes (*n* = 49) pre- and post-COVID-19 infection [7]. 

## 3. Results

### 3.1. Baseline Characteristics

We analyzed elite athletes (*n* = 43, average exercise time >10 h/week) of national football (*n* = 11), handball (*n* = 15), land hockey (*n* = 4), rowing (*n* = 3), badminton (*n* = 9), and swimming (*n* = 1) teams. Recreational athletes (*n* = 40, average exercise time >4 h/week) who mainly performed endurance sports were used as a control group (running *n* = 19, cycling *n* = 14); only a minority (*n* = 7) conducted cross-fit and bodybuilding. No premature exercise termination or adverse events during CPET occurred.

Three elite athletes were excluded from CPET testing due to elevated troponin. However, none of them displayed signs of (peri-) myocarditis on a subsequent MRI. As the last exercise had been performed >48 h before troponin testing, a diagnosis of “myocardial injury” was made, and the athletes were recommended to refrain from exercise. In two out of three participants, their troponin levels normalized within two weeks, while an elevated level persisted in one elite athlete even after three months and an additional normal MRI (see workflow; Figure 1).

Elite athletes were significantly younger (21.9 ± 3.7 years vs. 34.7 ± 13.0 years; *p* < 0.001) and were predominantly male (86.0% vs. 52.5%; *p* = 0.001). There was no clinically relevant differences in laboratory values (Table 1). Despite the left atrial volume index, elite athletes displayed higher values for left and right ventricular dimensions, but there was no difference in the left ventricular ejection fraction or tricuspid annular plane systolic excursion (TAPSE) as a surrogate for right ventricular function (Table 1). Neither the number of vaccinated patients nor the number of those who received vaccinations differed between groups. Additionally, time since the last COVID-19 vaccination did not differ between the groups (Table 1). During CPET, no abnormal electrocardiographic findings occurred in both groups.

### 3.2. CPET Outcome Variables

Elite athletes showed higher values for almost all the CPET parameters (Table 2). Chronotropic incompetence, defined as a failure to exceed 80% of the predicted peak heart rate, was not found in recreational nor elite athletes.

**Figure 2:**
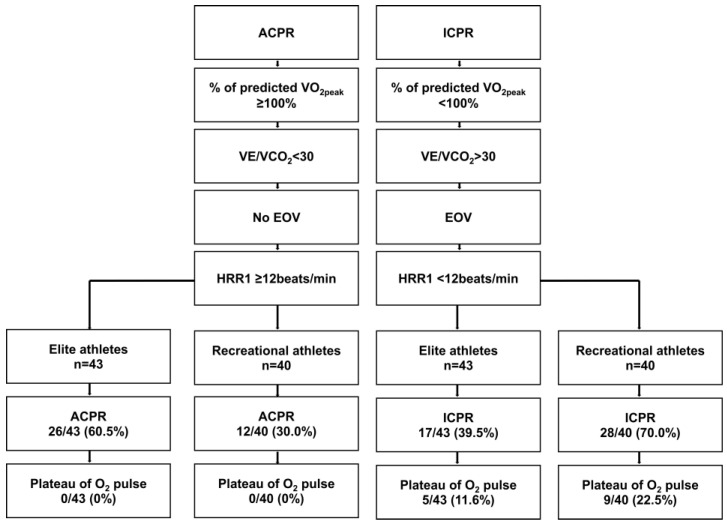
Definition and prevalence of the primary endpoint adequate cardiopulmonary response (ACPR) in elite and recreational athletes. ACPR is defined by the presence of all of the following criteria: (1) ≥100% of predicted peak oxygen consumption (VO2peak), (2) VE/VCO2 < 30, (3) no presence of exercise oscillatory ventilation (EOV), (4) heart rate recovery 1 min after exercise termination (HRR1) ≥ 12 beats/min. ICPR: inadequate cardiopulmonary response, which is defined by the absence of at least one criterion of ACPR. EOV: exercise oscillatory ventilation. HRR1: heart rate recovery one minute following exercise termination. Plateau of O2 pulse was defined as a deflection of the curve from one time point to the next.

ICPR occurred more frequently in recreational athletes compared to elite athletes (70.0% vs. 39.5%; *p* = 0.005, Figure 2). This difference was mainly driven by a larger proportion of recreational athletes with VE/VCO2 > 30 (35.0% vs. 2.3%, *p* < 0.001). EOV (22.5% vs. 14.0%, *p* = 0.234), predVO2peak <100% (50.0% vs. 32.6%, *p* = 0.082), and HRR1 < 12 beats/min (7.5% vs. 0%, *p* = 0.108) did not have a major impact on the higher number of ICPR in recreational athletes. Interestingly, the plateauing of O2 pulse occurred in all patients with ICPR both in elite and recreational athletes. There was no between-group difference (11.6% vs. 22.5%, *p* = 0.152, Figure 2).

The resting heart rate, peak systolic blood pressure, and peak performance indices of O2 pulse and peak minute ventilation differed between ACPR and ICPR (Table 3).

We also compared sex-specific differences in the ICPR response among CPET variables and only found the OUFES, minute ventilation, and peak performance to differ significantly, while VO2peak did not differ (Table 4).

The regression analysis showed a significant impact of sports category and ACPR on the OUES and VO2peak. Age was a confounder for both variables, but sex turned out to be a confounder only for the OUES. ACPR was associated with ΔHR, with age as a major confounder (Table A1).

**Figure 3:**
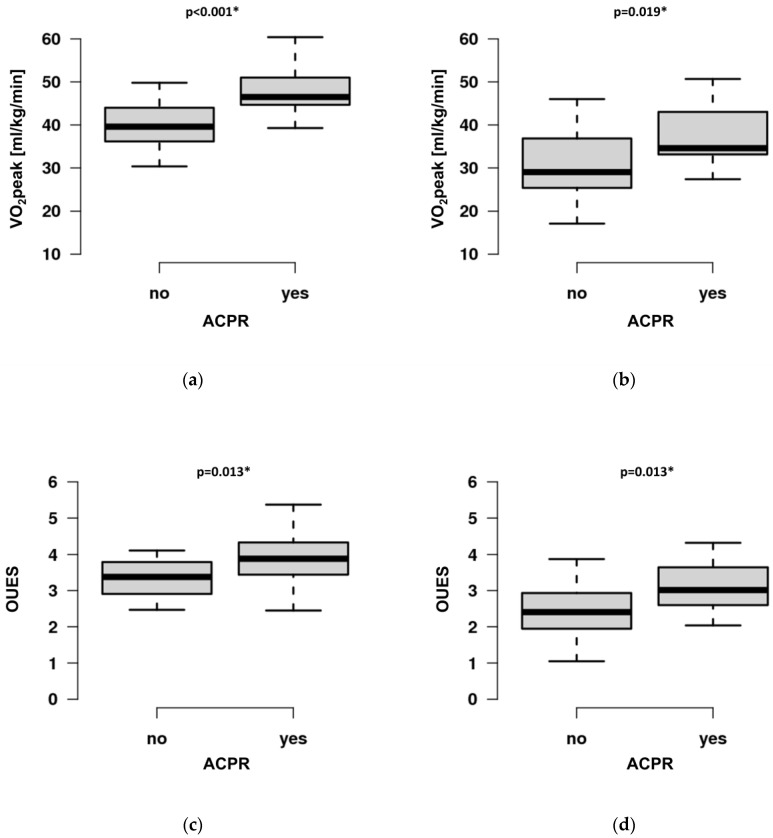

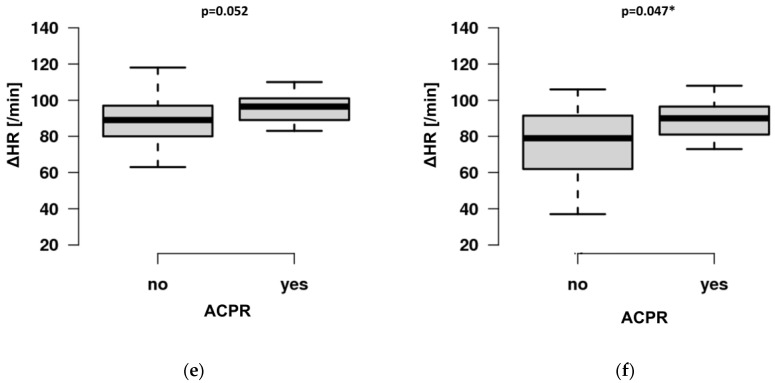
Comparison of selected variables of cardiopulmonary exercise testing (CPET) in elite (left) and recreational (right) athletes depending on adequate cardiopulmonary response (ACPR). ACPR is defined by a CPET performance of (1) ≥100% of predicted peak oxygen consumption (VO2peak), (2) VE/VCO2 < 30, (3) no presence of exercise oscillatory ventilation (EOV), (4) heart rate recovery 1 min after exercise termination ≥ 12 beats/min (HRR1). (**a**): VO2peak [mL/kg/min] in elite athletes. (**b**): VO2peak in recreational athletes. (**c**): Oxygen uptake efficiency slope (OUES) in elite athletes. (**d**): OUES in recreational athletes. (**e**): ΔHR (difference between resting and peak heart rate, /min) in elite and (**f**): recreational athletes. Significance is depicted with an asterisk.

Post hoc analysis revealed higher VO2peak (CI 4.10–10.60, *p* < 0.001 and CI 1.50–12.10, *p* = 0.019), OUES (CI 0.09–0.90, *p* = 0.013 and CI 0.17–1.17, *p* = 0.013), and ΔHR (CI 0–14, *p* = 0.052 and CI 0–23, *p* = 0.047) in ACPR both in elite and recreational athletes (Figure 3a–f).

All patients were diagnosed with PEM exceeding the necessary thresholds for steps 1 and 2 of the 10-item DSQ-PEM score. Although slight differences in numbers were observed across the items, the groups did not differ in any item (Table 5).

Based on the results of the DSQ-PEM items in the groups, we performed a post hoc comparison of those questions with differences between the groups. We combined questions 1–5 (Table 3) and compared the CPET variables of those participants exceeding the threshold for diagnosing PEM with those of those who did not. Although the hypothesis was generated based on a moderate sample size, we found that exceeding the threshold was associated with lower VO2peak (*p* = 0.023 and *p*= 0.031), ΔO2pulse (*p* = 0.024 and *p* = 0.045), O2pulsmax (*p* = 0.021 and *p* = 0.036), O2pulse25 (*p* = 0.034 and *p* = 0.034), O2pulse50 (*p* = 0.022 and 0.032), and O2pulse75 (*p* = 0.034 and 0.046) in elite and recreational athletes. Additionally, those exceeding the threshold displayed a higher resting heart rate (HRmin) (*p* = 0.034 and *p* = 0.029), higher VE/VCO2 (*p* = 0.025 and *p* = 0.019), as well as higher EqO2 at VT1 (*p* = 0.048 and *p* = 0.041) in elite as well as recreational athletes.

## 4. Discussion

### 4.1. CPET Performance of Athletes with PEM

Although there is no general recommendation to perform CPET in asymptomatic athletes upon RTP examination [32,33], CPET may be beneficial in cases of the persistence of exercise intolerance and PEM after COVID-19 infection for objectively revealing cardiopulmonary limitations. All of our participants reported exercise intolerance and were diagnosed with PEM according to the validated 10-item DSQ-PEM [13]. We did not observe ACPR in all of the highly trained recreational and elite athletes, which may signify underlying disease or incomplete recovery in the form of long-COVID-19 syndrome. This correlates with the recent findings of long-COVID-19 patients demonstrating PEM in 58.7% of the study population [11]. The deterioration of symptoms following exertional exercise is a hallmark of PEM and may have a detrimental effect on elite and recreational athletes who exercise on a regular basis. Identifying athletes with PEM who do not show ACPR, especially when displaying the plateauing of O2 pulse and VE/VCO2 > 30, may represent a higher-risk population for long-term sequelae of COVID-19. Combining DSQ-PEM screening and CPET in athletes with persistent exercise intolerance may aid in prescribing adequate and safe exercise training or even recommending refraining from exercise to prevent adverse events. Although no data have been supplied to demonstrate adverse events in athletes if exercise is continued, a higher risk could be expected. It has been shown that long-COVID-19 and PASC display persistent inflammatory activity [9,32]. Inflammation is associated with a higher rate of cardiac arrhythmias, such as atrial fibrillation and ectopies [34], which may further decrease exercise capacity or may even lead to the premature development of heart failure [32]. Further research is needed to guide athletes and coaches in return-to-play decisions and the tailoring of training intensity and duration.

ICPR was mainly driven by VE/VCO2 > 30 as a marker of inefficient ventilation and may thus be a promising parameter for quantifying persistent exercise limitations following COVID-19 infection. This is in accordance with previous reports that showed that COVID-19 leads to hyperventilation during recovery [3,5] and may accompany the development of long-COVID-19 syndrome, which is believed to manifest early in the recovery process [35]. A recent report on young (24.0 ± 4.5 years) male volleyball athletes following COVID-19 infection [36] demonstrated a VO2peak value comparable to that of our elite athletes (44.1 ± 3.4 mL/kg/min vs. 44.8 ± 6.8 mL/kg/min); however, the subjects in that study had considerably higher VE/VCO2 (32.6 ± 2.8 vs. 24.5 ± 2.4). These values are even higher than those detected in our recreational athletes (28.4 ± 5.4). As these were first-division (elite) volleyball players, disease severity and recovery may be more severe than in our population.

Importantly, all participants with the plateauing of O2 pulse displayed ICPR, both in elite and recreational athletes. The plateauing of O2 pulse has been described as a short-term aftermath of COVID-19 infection and as a correlate of impaired peripheral oxygen extraction [3]. Thus, it may also serve as a valuable marker for detecting exercise limitations in athletes with PEM at risk of developing long-COVID-19. In the future, this semi-quantitative approach may be enriched by an automated algorithm which may be more precise and provide additional information on O2 pulse kinetics during exercise [37]. Diminished O2 pulsemax has been shown to occur in ambulatory post-COVID-19 patients [3], but this was not demonstrated by our athletes.

Athletes with ACPR demonstrated higher VO2peak, OUES, and ΔHR compared to ICPR. As expected, elite athletes performed better than recreational athletes in almost every CPET variable (Table 2, Figure 3). Recreational athletes were older, with a higher percentage of females. However, the implemented CPET variables and thresholds from ESC recommendations (predVO2peak, VE/VCO2, EOV, HRR1) do not depend on these potential confounders. Importantly, although we found a significant difference between groups in VO2peak, predVO2peak did not differ. Thus, predVO2peak rather than VO2peak should be used to analyze exercise limitations in athletes.

In participants with ICPR, we observed higher HRmin, lower peak systolic pressure, lower O2 pulse indices, as well as lower OUES and VO2peak. In particular, higher HRmin (and, thus, lower ΔHR with a constant peak heart rate), as a sign of an increased sympathetic tone, and lower O2 pulse indices, as markers for reduced peripheral oxygen extraction, may serve as additional variables for displaying reduced exercise capacity in long-COVID-19 athletes with PEM. In females with ICPR, we observed lower OUES but not VO2peak compared to males, which may signify that exertion-independent OUES may even be a more suitable variable than the widely studied maximal variable VO2peak. Further studies in athletes with PEM following COVID-19 infection are warranted to analyze these variables and their prognostic impact on the time until return-to-sports.

### 4.2. The Benefit of CPET in Athletes with PEM

Although no cut-off values for abnormal CPET in athletes exist, falling below the threshold values suggested by the ESC for apparently healthy individuals may herald a prolonged reconvalescence in athletes and prompt coaches to delay the onset of high-intensity training. The availability of CPET data on elite athletes is very limited [36], and current return-to-play (RTP) recommendations do not require standard CPET exams in asymptomatic athletes [32], mainly due to the considerable effort and expected high “number needed to test” needed in order to reveal the underlying pathology. However, it appears mandatory to perform CPET in athletes with PEM and persistent exercise intolerance to provide additional objective criteria for the definition of long-COVID-19 syndrome [38]. Post/long-COVID-19 is defined as the onset of COVID-19 with symptoms that last for at least two months and cannot be explained by an alternative diagnosis three months after a diagnosis of COVID-19; insufficient objective data on exercise limitations exist in athletes [39]. Furthermore, no data are available on a potential detrimental effect of continued high-intensity training in (both elite and recreational) athletes with PEM and persistent exercise intolerance, which may foster continued inflammation and viral persistence, which has been shown in long-COVID-19 patients [9,32]. 

In our study, the necessary thresholds for diagnosing PEM were reached in all participants in both elite and recreational athletes. Although this is only hypothesis-generating due to the limited size of the sample, we found that exceeding the threshold in those questions with differences between groups (questions 1–5, Table 5) was associated with decreased performance (VO2peak) and reduced oxygen extraction (O2 pulse). The latter could be attributed to residual endotheliitis as a hallmark of COVID-19. Our data support recent suggestions that studies should be conducted that analyze both the CPET response to exercise in persistent exercise limitations following COVID-19 infection as well as the immunological profile of these individuals [9]. This will provide additional insights into the pathophysiology of the disease and may also have significant implications for athletic training. Continuing high-intensity training with underlying, ongoing inflammation may lead to negative long-term effects for athletes, such as the development of cardiac arrhythmias and the maintenance of hampered oxygen delivery to mitochondria. Thus, future studies should apply biomarkers, immunological responses, the assessment of (validated) subjective scores, such as the DSQ-PEM, and CPET to attain a holistic image of exercise impairment and may be useful in tailoring individual RTP pathways for athletes by guiding the duration and intensity of training. However, not only athletes but also active individuals may benefit from this approach. It has been shown that VO2peak is considerably lower in patients who have been hospitalized (29.2 ± 0.3 mL/kg/min) due to COVID-19 infection (and even worse in ICU patients with a mean VO2peak value of 25.5 mL/kg/min) compared to those who were non-hospitalized (33.7 ± 7.0 mL/kg/min) [6]; VO2peak in the latter group was comparable to that of our recreational athletes. Additionally, VO2peak has recently been shown to be a reasonable marker for demonstrating exercise limitations after mild COVID-19 infection in endurance athletes compared to CPET before the infection (47.8 ± 7.8 mL/kg/min vs. 44.97 ± 7.00 mL/kg/min) [8]. However, the VO2peak findings from this study, which contained a similar sample size to that of our population (*n* = 49), must be interpreted with caution. (1) Pre-COVID-19 CPET testing was incorporated from a considerably long period, dating back to up to three years before the actual infection. Information on training progress or decline or additional diseases (mean age 39.9 ± 7.8 years) has not been provided during this period. (2) Treadmill as well as bicycle testing was performed, and VO2peak was compared independent of the modality. However, it has been demonstrated that VO2peak differs by at least 5% among modalities as a result of a higher degree of working musculature and energy expenditure during treadmill testing [40]. 

In our study, we used a combined approach of established CPET cut-off values for apparently healthy individuals [17], which are not expected to be crossed by athletes. However, 40% of elite and 70% of recreational athletes with PEM evidenced limitations, which provides substance for measurable exercise limitations. The population of participants with PEM and ACPR (60% of elite and 30% of recreational athletes) did not show limitations in exercise testing despite a high burden of stress. On the one hand, this may be explained by the fact that the DSQ-PEM has not been validated specifically for high-performance athletes after COVID-19 infection. On the other hand, other cognitive and emotional aspects in athletes may play a role; it has been shown that long-COVID-19 syndrome is associated with a decrease in mental health and reduced quality of sleep [41]. Patients without measurable CPET limitations and pathological DSQ-PEM scores could benefit from neuro-meditation programs, as these have been shown to improve mental health, depression, and anxiety in PASC [42]. Thus, athletes without CPET limitations and diagnosed PEM should be extensively screened for depression.

In our study, we did not find any athlete with chronotropic incompetence, which may be explained by the lack of apparent disease. We found a higher heart rate prior to exercise testing in patients exceeding the thresholds for questions 1–5 of the DSQ-PEM. Although PEM has been associated with tachycardia [11], this did not apply to our highly trained (elite) cohort. Elite athletes have a higher vagal tone compared to controls; thus, the elevation of the resting heart rate may not fulfill the criteria for tachycardia, but it should still be analyzed over time. We did not have prior data on the resting heart rate in our population to demonstrate an increase over time, which may express a higher sympathetic tone as a result of PEM.

We suggest combining the 10-item DSQ-PEM scoring with CPET to identify athletes at risk of long-COVID-19 syndrome and with a higher risk of long-term morbidity. Additional simultaneous immunological testing may even further refine our understanding of long-COVID-19 pathophysiology. Prospective trials are warranted to determine whether the presence of the plateauing of O2 pulse, VE/VCO2 > 30, as well as O2 pulse indices or a resting heart rate may represent a phenotype at a higher risk of developing long-COVID-19 syndrome.

### 4.3. Limitations

Our study has several limitations that must be considered. 

(1) This was a retrospective study with inherent methodological limitations. 

(2) Elite athletes all participated on an international level. Thus, no follow-up CPET study was available due to the upcoming start of the regular season. We also did not have previous CPET data from our athletes because meticulous CPET follow-up was started at our institution with the onset of this study. 

(3) As this was an investigational pilot study, we did not have a control group for elite and recreational athletes without prior COVID-19 infection.

(4) There may be sport-specific differences in exercise performance following COVID-19 infections. 

(5) The mode of exercise (bicycle) may influence the peak oxygen consumption and other derived variables. 

(6) Age and sex differed significantly between the groups and must be considered as relevant confounders, although predVO2peak corrects for these confounders. 

(7) The application of CPET cut-offs from ESC recommendations to assess cardiovascular risk has been validated for apparently healthy individuals but not for a population of athletes. 

(8) Athletes reported subjective exercise intolerance at the study inclusion, and PEM was diagnosed in all participants according to the DSQ-PEM. Although this score has been validated in a general cohort, no data are available on its validity in an athletic population. 

(9) No follow-up CPET is available for analyzing the resolution of objective limitations over time.

## 5. Conclusions

ACPR was not observed in all athletes with persistent subjective exercise intolerance and diagnosed PEM. In particular, higher VE/VCO2 and the plateauing of O2 pulse, but also lower O2 pulse indices and a higher resting heart rate, were associated with ICPR and may be useful for revealing athletes with more pronounced exercise limitations. Combining the 10-item DSQ-PEM with these CPET variables may help to better identify a phenotype of athletes with long-COVID-19 syndrome, which should trigger the adaptation of exercise intensities and may even delay return-to-sports recommendations in athletes.

## Figures and Tables

**Table 1:** Patient characteristics in elite and recreational athletes.

Medical History	Elite (*n* = 43)	Recreational (*n* = 40)	*p*-Value
Age (years)	21.9 ± 3.7	34.7 ± 13.0	*p* < 0.001 *
BMI (kg/m^2^)	23.7 ± 2.2	24.3 ± 3.7	*p* = 0.716
Women (%)	14.0 (6/43)	47.5 (19/40)	*p* = 0.001 *
Patients vaccinated (%)	42	37	*p* = 0.845
Vaccinations received	2.5 ± 0.4	2.2 ± 0.5	*p* = 0.345
Time since last vaccination (months)	3.4 ± 2.1	2.8 ± 1.9	*p* = 0.324
NTproBNP (pg/mL)	44.6 ± 0.25.1	50.2 ± 38.4	*p* = 0.481
Hemoglobin (g/dL)	15.1 ± 1.0	14.3 ± 1.2	*p* < 0.001 *
Troponin (ng/L) a	4.8 ± 5.7	3.4 ± 1.7	*p* = 0.008 *
D-Dimer (mg/L)	0.3 ± 0.1	0.3 ± 0.2	*p* = 0.268
Ferritin (mg/L)	90.0 ± 54.2	81.0 ± 50.6	*p* = 0.581
CRP (mg/dL)	0.3 ± 0.1	0.2 ± 0.1	*p* < 0.001 *
LVEF (%)	59.6 ± 4.8	59.2 ± 4.6	*p* = 0.330
LAVI (mL/m^2^)	28.3 ± 9.2	24.5 ± 5.6	*p* = 0.073
LVMI (g/m^2^)	93.5 ± 18.9	81.0 ± 25.4	*p* = 0.001 *
LVEDD (mm)	51.6 ± 5.1	49.1 ± 5.0	*p* = 0.024 *
TAPSE (mm)	25.9 ± 3.9	25.6 ± 4.3	*p* = 0.945
sPAP (mmHg)	17.3 ± 5.4	17.6 ± 5.4	*p* = 0.434
RV basal (mm)	38.6 ± 4.5	33.5 ± 6.1	*p* < 0.001 *
RV mid (mm)	29.3 ± 4.8	25.6 ± 6.0	*p* = 0.002 *

BMI: Body mass index [kg/m^2^]; NTproBNP: N-terminal prohormone of brain natriuretic peptide; CRP: C-reactive protein; LVEF: left ventricular ejection fraction; LAVI: left atrial volume index; LVEDD: left ventricular end-diastolic diameter; LVMI: left ventricular mass index; sPAP: systolic pulmonary artery pressure; RV basal: basal diameter of right ventricle; RV mid: midventricular diameter of the right ventricle; TAPSE: tricuspid annular plane systolic excursion. Mean values are depicted with standard deviations; absolute values are provided in round brackets. * Significance at alpha < 0.05. Cut-off values for high-sensitivity troponin < 35 ng/L.

**Table 2:** Outcome variables of cardiopulmonary exercise testing (CPET) in elite and recreational athletes.

CPET Variables	Elite (*n* = 43)	Recreational (*n* = 40)	*p*-Value
HRmin (beats/min)	84.4 ± 14.3	93.0 ± 16.9	*p* = 0.025 *
HRmax (beats/min)	180.2 ± 12.5	172.3 ± 17.1	*p* = 0.028 *
HRR1 (beats/min)	28.5 ± 7.7	28.7 ± 14.1	*p* = 0.587
RRsysmax (mmHg)	194.1 ± 30.4	179.5 ± 27.3	*p* = 0.047 *
RRdiamax (mmHg)	90.0 ± 16.8	89.6 ± 14.4	*p* = 0.906
P max (W)	302.9 ± 45.5	204.6 ± 59.3	*p* < 0.001 *
VO2peak (mL/kg/min)	44.8 ± 6.8	32.7 ± 7.9	*p* < 0.001 *
% of VO2pred	107.4 ± 17.7	101.3 ± 24.5	*p* = 0.204
VE (L)	117.9 ± 26.5	93.4 ± 24.4	*p* < 0.001 *
VE/VCO2	24.5 ± 2.4	28.4 ± 5.4	*p* < 0.001 *
BR (%)	23.7 ± 17.5	26.3 ± 16.6	*p* = 0.542
Plateau of O2 pulse (%)	11.6 (5/43)	22.5 (9/40)	*p* = 0.152
O2 pulserest (mL/beat/kg × 100)	10.4 ± 2.2	9.2 ± 2.7	*p* = 0.012 *
O2 pulsemax (mL/beat/kg × 100)	24.2 ± 3.9	18.6 ± 4.4	*p* < 0.001 *
ΔO2pulse	13.8 ± 4.0	9.4 ± 3.4	*p* < 0.001 *
O2 pulse25 (mL/beat/kg × 100)	16.5 ± 3.3	13.4 ± 3.2	*p* < 0.001 *
O2 pulse50 (mL/beat/kg × 100)	20.5 ± 3.8	16.4 ± 4.0	*p* < 0.001 *
O2 pulse75 (mL/beat/kg × 100)	22.8 ± 4.1	18.1 ± 4.1	*p* < 0.001 *
EqO2 at VT1	19.0 ± 2.3	21.1 ± 2.7	*p* < 0.001 *
OUES	3.7 ± 0.7	2.6 ± 0.7	*p* < 0.001 *
EOV	14.0 (6/43)	22.5 (9/40)	*p* = 0.234
ACPR ≥100% of predVO2peak VE/VCO2 < 30 No EOV HRR1≥ 12 beats/min	26/43 (60.5%) 29/43 (67.4%) 42/43 (97.7%) 37/43 (86.0%) 43/43 (100.0%)	12/40 (30.0%) 20/40 (50%) 26/40 (65%) 31/40 (77.5%) 37/40 (92.5%)	*p* = 0.005 * p = 0.082 *p* < 0.001 * p = 0.234 *p* = 0.108

Outcome variables of cardiopulmonary exercise testing (CPET) in elite and recreational athletes. HRmin: minimal heart rate; HRmax: maximal heart rate at peak exercise; HRR1: heart rate recovery 1 min after exercise termination; RRsysmax: systolic pressure at peak exercise; RRdiamax: diastolic pressure at peak exercise; Pmax: maximal power achieved; VO2peak: peak oxygen consumption; ≥100% of predVO2peak: number of participants ≥ 100% of predicted peak oxygen consumption according to the Hansen–Wasserman equation; VE/VCO2: ventilatory efficiency slope; O2 pulsemax: O2 pulse at peak exercise related to body weight; O2 pulserest: O2 pulse at rest related to body weight; O2 pulse25: O2 pulse at 25% of exercise time related to body weight; O2 pulse50: O2 pulse at 50% of exercise time related to body weight; O2 pulse75: O2 pulse at 75% of exercise time related to body weight; ΔO2pulse: difference between O2 pulse at rest and peak exercise; EqO2 at VT1: oxygen equivalent at the first ventilatory threshold; OUES: oxygen uptake efficiency slope; EOV: exercise oscillatory ventilation; BR: breathing reserve; ACPR: adequate cardiopulmonary response was defined by the presence of all of the following criteria: (1) peak oxygen consumption ≥ 100% of predicted (predVO2peak), (2) VE/VCO2 < 30, (3) no presence of exercise oscillatory ventilation (EOV), and (4) heart rate recovery of ≥12 beats/minute 1 min after exercise termination (HRR1). Mean values are depicted with standard deviations; absolute values are shown in round brackets. Significance is denoted with an asterisk at alpha < 0.05.

**Table 3:** CPET variables depending on cardiopulmonary response.

CPET Variables	ACPR (*n* = 43)	ICPR (*n* = 40)	*P*-Value
HRmin (beats/min)	84.3 ± 13.7	93.1 ± 16.8	*p* = 0.017 *
HRmax (beats/min)	179.2 ± 11.3	174.0 ± 17.9	*p* = 0.320
RRsysmax (mmHg)	194.9 ± 30.9	180.4 ± 27.2	*p* = 0.046 *
RRdiamax (mmHg)	87.0 ± 15.5	92.2 ± 15.5	*p* = 0.179
Pmax (W)	291.8 ± 64.7	224.8 ± 63.4	*p* < 0.001 *
VO2peak (mL/kg/min)	44.4 ± 8.0	34.3 ± 8.2	*p* < 0.001 *
VE (L)	117.6 ± 31.8	96.3 ± 22.6	*p* = 0.004 *
BR (%)	23.6 ± 15.6	26.1 ± 18.2	*p* = 0.567
O2 pulserest (mL/beat/kg × 100)	10.4 ± 2.5	9.4 ± 2.5	*p* < 0.001 *
O2 pulsemax (mL/beat/kg × 100)	24.2 ± 4.3	19.2 ± 4.4	*p* < 0.001 *
ΔO2pulse	13.9 ± 4.2	9.8 ± 3.5	*p* < 0.001 *
O2 pulse25 (mL/beat/kg × 100)	16.5 ± 3.4	13.8 ± 3.3	*p* < 0.001 *
O2 pulse50 (mL/beat/kg × 100)	20.5 ± 4.1	16.9 ± 3.9	*p* < 0.001 *
O2 pulse75 (mL/beat/kg × 100)	23.0 ± 4.4	18.5 ± 4.0	*p* < 0.001 *
EqO2 at VT1	19.6 ± 2.7	20.3 ± 2.7	*p* = 0.221
OUES	3.6 ± 0.8	2.8 ± 0.8	*p* < 0.001 *

CPET: cardiopulmonary exercise testing; HRmin: minimal heart rate; HRmax: maximal heart rate at peak exercise; RRsysmax: systolic pressure at peak exercise; RRdiamax: diastolic pressure at peak exercise; Pmax: maximal power achieved; VO2peak: peak oxygen consumption; O2 pulsemax: O2 pulse at peak exercise related to body weight; O2 pulserest: O2 pulse at rest related to body weight; O2 pulse25: O2 pulse at 25% of exercise time related to body weight; O2 pulse50: O2 pulse at 50% of exercise time related to body weight; O2 pulse75: O2 pulse at 75% of exercise time related to body weight; ΔO2pulse: difference between O2 pulse at rest and peak exercise; EqO2 at VT1: oxygen equivalent at the first ventilatory threshold; OUES: oxygen uptake efficiency slope; ACPR: adequate cardiopulmonary response was defined by the presence of all of the following criteria: (1) peak oxygen consumption (VO2peak) ≥ 100% of predicted (predVO2peak), (2) VE/VCO2 < 30, (3) no presence of exercise oscillatory ventilation (EOV), and (4) heart rate recovery (HRR) of ≥12 beats/minute 1 min after exercise termination. ICPR: inadequate cardiopulmonary response was defined as a lack of one of the criteria for ACPR. Mean values are depicted with standard deviations; absolute values are shown in round brackets. Significance is denoted with an asterisk at alpha < 0.05.

**Table 4:** Sex-specific differences in ICPR.

CPET Variables	Female (*n* = 12)	Male (*n* = 33)	*p*-Value
HRmin (beats/min)	97.3 ± 17.8	91.4 ± 16.0	*p* = 0.292
HRmax (beats/min)	171.3 ± 24.5	176.0 ± 24.5	*p* = 0.443
RRsysmax (mmHg)	170.7 ± 21.8	182.9 ± 21.8	*p* = 0.175
RRdiamax (mmHg)	91.8 ± 9.6	91.7 ± 17.1	*p* = 0.978
Pmax (W)	163.3 ± 42.4	249.3 ± 56.3	*p* < 0.001 *
VO2peak (mL/kg/min)	32.2 ± 7.4	35.8 ± 8.7	*p* = 0.210
VE (L)	76.1 ± 18.9	104.5 ± 19.8	*p* < 0.001 *
BR (%)	22.4 ± 15.5	26.9 ± 18.8	*p* = 0.460
O2 pulserest (mL/beat/kg × 100)	10.1 ± 3.0	9.3 ± 2.3	*p* = 0.317
O2 pulsemax (mL/beat/kg × 100)	18.3 ± 5.0	19.8 ± 4.2	*p* = 0.316
ΔO2pulse	8.2 ± 3.1	10.6 ± 3.7	*p* = 0.056
O2 pulse25 (mL/beat/kg × 100)	13.9 ± 3.9	13.9 ± 3.1	*p* = 0.983
O2 pulse50 (mL/beat/kg × 100)	16.4 ± 4.7	17.2 ± 3.7	*p* = 0.547
O2 pulse75 (mL/beat/kg × 100)	18.2 ± 4.6	18.8 ± 3.8	*p* = 0.637
EqO2 at VT1	21.2 ± 2.8	19.8 ± 2.6	*p* = 0.172
OUES	2.2 ± 0.7	3.0 ± 0.7	*p* = 0.001 *

CPET: Cardiopulmonary exercise testing; HRmin: minimal heart rate; HRmax: maximal heart rate at peak exercise; RRsysmax: systolic pressure at peak exercise; RRdiamax: diastolic pressure at peak exercise; Pmax: maximal power achieved; VO2peak: peak oxygen consumption; O2 pulsemax: O2 pulse at peak exercise related to body weight; O2 pulserest: O2 pulse at rest related to body weight; O2 pulse25: O2 pulse at 25% of exercise time related to body weight; O2 pulse50: O2 pulse at 50% of exercise time related to body weight; O2 pulse75: O2 pulse at 75% of exercise time related to body weight; ΔO2pulse: difference between O2 pulse at rest and peak exercise; EqO2 at VT1: oxygen equivalent at the first ventilatory threshold; OUES: oxygen uptake efficiency slope; ACPR: adequate cardiopulmonary response was defined by the presence of all of the following criteria: (1) peak oxygen consumption (VO2peak) ≥ 100% of predicted (predVO2peak), (2) VE/VCO2 < 30, (3) no presence of exercise oscillatory ventilation (EOV), and (4) heart rate recovery (HRR) of ≥12 beats/minute 1 min after exercise termination. ICPR: inadequate cardiopulmonary response was defined as a lack of one of the criteria for ACPR. Mean values are depicted with standard deviations; absolute values are shown in round brackets. Significance is denoted with an asterisk at alpha < 0.05.

**Table 5:** Post-exertional malaise in the study population.

10-Item DSQ-PEM	Elite (*n* = 43)	Recreational (*n* = 40)	*p*-Value
Diagnosed PEM (%) in scoring step 1	43 (100.0)	40 (100.0)	*p* > 0.999
Diagnosed PEM (%) in scoring step 2	43 (100.0)	40 (100.0)	*p* > 0.999
1. Dead, heavy feeling after launching of exercise	40 (93.0)	35 (87.5)	*p* = 0.564
2. Soreness of fatigue the next day after non-strenuous, everyday activity	28 (65.1)	24 (60.0)	*p* = 0.346
3. Mentally tired after low effort	31 (72.1)	27 (67.5)	*p* = 0.397
4. A minimum amount of exercise leads to physical exhaustion	39 (90.7)	36 (90.0)	*p* = 0.897
5. A feeling of sickness after mild physical activity	25 (58.1)	21 (52.5)	*p* = 0.721
6. A lack of recovery within 2 h after exhausting activity	43 (100.0)	40 (100.0)	*p* > 0.999
7. A worsening of fatigue after minimal physical effort	43 (100.0)	40 (100.0)	*p* > 0.999
8. A worsening of fatigue after minimal mental effort	43 (100.0)	40 (100.0)	*p* > 0.999
9. Feeling worse after activities and persistence for at least 14 h	43 (100.0)	40 (100.0)	*p* > 0.999
10. Evading exercise, as it makes symptoms worse	2 (4.7)	3 (7.5)	*p* = 0.351

10-item DSQ-PEM: 10-item DePaul Symptom Questionnaire—Post-Exertional Malaise; PEM: post-exertional malaise. In the first scoring step (questions 1–5), a threshold of 1 or more must be exceeded for the first five DSQ-PEM items. A score of 2–4 for frequency (half the time, most of the time, all of the time) together with a score of 2–4 for severity (moderate, severe, or very severe) for the same item was considered diagnostic for PEM. The supplementary second part (questions 6–10) contains questions on exercise exacerbation, quick recovery, and PEM duration. PEM requires an answer of “yes” for items 7 and 8, while a response of ≥14 h is required for item 9. Data are presented for the numbers and percentages (in round brackets) of items exceeding the necessary threshold to be diagnostic for PEM. All participants in both groups were diagnosed with PEM. Mean values are depicted with standard deviations; absolute values are provided in round brackets.

## Data Availability

Data will be made available by the corresponding author on reasonable request.

## Appendix A

**Table A1:** Multivariable linear regression model for selected variables of cardiopulmonary exercise testing.

Variable	Estimate	Standard Error	R2	*p*-Value
VO2peak Intercept Sports category ACPR Age	47.409 −6.009 6.873 −0.310	1.979 1.619 1.367 0.070	R2 = 0.614	*p* < 0.001 * *p* = 0.001 * *p* < 0.001 * *p* < 0.001 *
OUES Intercept Sports category ACPR Age Female	3.611 −0.388 0.748 −0.014 −0.750	0.190 0.167 0.137 0.007 0.152	R2 = 0.581	*p* < 0.001 * *p* = 0.023 * *p* < 0.001 * *p* = 0.045 * *p* < 0.001 *
ΔHR Intercept ACPR Age	101.052 10.943 −0.0667	5.113 3.436 0.1518	R2 = 0.296	*p* < 0.001 * *p* = 0.002 * *p* < 0.001 *

Multivariable linear regression model for selected variables of cardiopulmonary exercise testing (CPET) for estimating the impact of sports category (elite vs. recreational athlete) and adequate cardiorespiratory response during exercise. Age and sex were considered as confounders. ACPR is defined by a CPET performance of all of the following: (1) ≥100% of predicted peak oxygen consumption (predVO2peak), (2) VE/VCO2 < 30, (3) no presence of exercise oscillatory ventilation (EOV), (4) heart rate recovery 1 min after exercise termination ≥ 12 beats/min (HRR1). ΔHR: difference between resting and peak heart rate. OUES: oxygen uptake efficiency slope. VO2peak: peak oxygen consumption. Significance is denoted with an asterisk at alpha < 0.05. R2 is provided for each variable.

## References

[1] Jennifer K., Shirley S.B.D., Avi P., Daniella R.C., Naama S.S., Anat E.Z., Miri M.R. (2023). Post-acute sequelae of COVID-19 infection. Prev. Med. Rep..

[2] Wilson M.G., Hull J.H., Rogers J., Pollock N., Dodd M., Haines J., Harris S., Loosemore M., Malhotra A., Pieles G. (2020). Cardiorespiratory considerations for return-to-play in elite athletes after COVID-19 infection: A practical guide for sport and exercise medicine physicians. Br. J. Sport. Med..

[3] Evers G., Schulze A.B., Osiaevi I., Harmening K., Vollenberg R., Wiewrodt R., Pistulli R., Boentert M., Tepasse P.R., Sindermann J.R. (2022). Sustained Impairment in Cardiopulmonary Exercise Capacity Testing in Patients after COVID-19: A Single Center Experience. Can. Respir. J..

[4] Skjørten I., Ankerstjerne O.A.W., Trebinjac D., Brønstad E., Rasch-Halvorsen Ø., Einvik G., Lerum T.V., Stavem K., Edvardsen A., Ingul C.B. (2021). Cardiopulmonary exercise capacity and limitations 3 months after COVID-19 hospitalisation. Eur. Respir. J..

[5] Baratto C., Caravita S., Faini A., Perego G.B., Senni M., Badano L.P., Parati G. (2021). Impact of COVID-19 on exercise pathophysiology: A combined cardiopulmonary and echocardiographic exercise study. J. Appl. Physiol..

[6] Lemos M.M., Cavalini G.R., Pugliese Henrique C.R., Perli V.A.S., de Moraes Marchiori G., Marchiori L.L.M., Sordi A.F., Franzói de Moraes S.M., de Paula Ramos S., Valdés-Badilla P. (2022). Body composition and cardiorespiratory fitness in overweight or obese people post COVID-19: A comparative study. Front. Physiol..

[7] Silva F.B.D., Fonseca B., Domecg F., Facio M.R., Prado C., Toledo L., Tuche W. (2021). Athletes health during pandemic times: Hospitalization rates and variables related to COVID-19 prevalence among endurance athletes. Int. J. Cardiovasc. Sci..

[8] Śliż D., Wiecha S., Gąsior J.S., Kasiak P.S., Ulaszewska K., Lewandowski M., Barylski M., Mamcarz A. (2023). Impact of COVID-19 Infection on Cardiorespiratory Fitness, Sleep, and Psychology of Endurance Athletes-CAESAR Study. J. Clin. Med..

[9] Deng M.C. (2023). An exercise immune fitness test to unravel mechanisms of Post-Acute Sequelae of COVID-19. Expert Rev. Clin. Immunol..

[10] Buonsenso D., Di Giuda D., Sigfrid L., Pizzuto D.A., Di Sante G., De Rose C., Lazzareschi I., Sali M., Baldi F., Chieffo D.P.R. (2021). Evidence of lung perfusion defects and ongoing inflammation in an adolescent with post-acute sequelae of SARS-CoV-2 infection. Lancet Child Adolesc. Health.

[11] Twomey R., DeMars J., Franklin K., Culos-Reed S.N., Weatherald J., Wrightson J.G. (2022). Chronic Fatigue and Postexertional Malaise in People Living with Long COVID: An Observational Study. Phys. Ther..

[12] Chu L., Valencia I.J., Garvert D.W., Montoya J.G. (2018). Deconstructing post-exertional malaise in myalgic encephalomyelitis/chronic fatigue syndrome: A patient-centered, cross-sectional survey. PLoS ONE.

[13] Cotler J., Holtzman C., Dudun C., Jason L.A. (2018). A Brief Questionnaire to Assess Post-Exertional Malaise. Diagnostics.

[14] NINDS Common Data Elements (CDE) Group Post-Exertional Malaise Subgroup Summary. Myalgic Encephalomyelitis/Chronicfatiguesyndrome. https://www.commondataelements.ninds.nih.gov/sites/nindscde/files/Doc/MECFS/PEM_Subgroup_Summary.pdf.

[15] Phelan D., Kim J.H., Elliott M.D., Wasfy M.M., Cremer P., Johri A.M., Emery M.S., Sengupta P.P., Sharma S., Martinez M.W. (2020). Screening of Potential Cardiac Involvement in Competitive Athletes Recovering From COVID-19: An Expert Consensus Statement. JACC Cardiovasc. Imaging.

[16] Symanski J.D., Tso J.V., Phelan D.M., Kim J.H. (2022). Myocarditis in the Athlete: A Focus on COVID-19 Sequelae. Clin. Sport. Med..

[17] Guazzi M., Arena R., Halle M., Piepoli M.F., Myers J., Lavie C.J. (2018). 2016 focused update: Clinical recommendations for cardiopulmonary exercise testing data assessment in specific patient populations. Eur. Heart J..

[18] Pelliccia A., Sharma S., Gati S., Bäck M., Börjesson M., Caselli S., Collet J.P., Corrado D., Drezner J.A., Halle M. (2021). 2020 ESC Guidelines on sports cardiology and exercise in patients with cardiovascular disease. Eur. Heart J..

[19] McKinney J., Velghe J., Fee J., Isserow S., Drezner J.A. (2019). Defining Athletes and Exercisers. Am. J. Cardiol..

[20] Wu X., Liu X., Zhou Y., Yu H., Li R., Zhan Q., Ni F., Fang S., Lu Y., Ding X. (2021). 3-month, 6-month, 9-month, and 12-month respiratory outcomes in patients following COVID-19-related hospitalisation: A prospective study. Lancet Respir. Med..

[21] Lang R.M., Badano L.P., Mor-Avi V., Afilalo J., Armstrong A., Ernande L., Flachskampf F.A., Foster E., Goldstein S.A., Kuznetsova T. (2015). Recommendations for cardiac chamber quantification by echocardiography in adults: An update from the American Society of Echocardiography and the European Association of Cardiovascular Imaging. Eur. Heart J.-Cardiovasc. Imaging.

[22] Guazzi M., Adams V., Conraads V., Halle M., Mezzani A., Vanhees L., Arena R., Fletcher G.F., Forman D.E., Kitzman D.W. (2012). EACPR/AHA Scientific Statement. Clinical recommendations for cardiopulmonary exercise testing data assessment in specific patient populations. Circulation.

[23] Hansen J.E., Sue D.Y., Wasserman K. (1984). Predicted values for clinical exercise testing. Am. Rev. Respir. Dis..

[24] Kremser C.B., O’Toole M.F., Leff A.R. (1987). Oscillatory hyperventilation in severe congestive heart failure secondary to idiopathic dilated cardiomyopathy or to ischemic cardiomyopathy. Am. J. Cardiol..

[25] Laukkanen J.A., Araújo C.G.S., Kurl S., Khan H., Jae S.Y., Guazzi M., Kunutsor S.K. (2018). Relative peak exercise oxygen pulse is related to sudden cardiac death, cardiovascular and all-cause mortality in middle-aged men. Eur. J. Prev. Cardiol..

[26] Laukkanen J.A., Savonen K., Hupin D., Araújo C.G.S., Kunutsor S.K. (2021). Cardiorespiratory optimal point during exercise testing and sudden cardiac death: A prospective cohort study. Prog. Cardiovasc. Dis..

[27] Baba R., Nagashima M., Goto M., Nagano Y., Yokota M., Tauchi N., Nishibata K. (1996). Oxygen uptake efficiency slope: A new index of cardiorespiratory functional reserve derived from the relation between oxygen uptake and minute ventilation during incremental exercise. J. Am. Coll. Cardiol..

[28] Bhatia R., Cohen B.H., McNinch L.N. (2021). A novel exercise testing algorithm to diagnose mitochondrial myopathy. Muscle Nerve.

[29] Arena R., Myers J., Guazzi M. (2008). The clinical and research applications of aerobic capacity and ventilatory efficiency in heart failure: An evidence-based review. Heart Fail. Rev..

[30] A Language and Environment for Statistical Computing. R Foundation for Statistical Computing. https://www.R-project.org/.

[31] Cohen J. (1988). Statistical Power Analysis for the Behavioral Sciences.

[32] Gluckman T.J., Bhave N.M., Allen L.A., Chung E.H., Spatz E.S., Ammirati E., Baggish A.L., Bozkurt B., Cornwell W.K., Harmon K.G. (2022). 2022 ACC Expert Consensus Decision Pathway on Cardiovascular Sequelae of COVID-19 in Adults: Myocarditis and Other Myocardial Involvement, Post-Acute Sequelae of SARS-CoV-2 Infection, and Return to Play: A Report of the American College of Cardiology Solution Set Oversight Committee. J. Am. Coll. Cardiol..

[33] Halle M., Bloch W., Niess A.M., Predel H.G., Reinsberger C., Scharhag J., Steinacker J., Wolfarth B., Scherr J., Niebauer J. (2021). Exercise and sports after COVID-19-Guidance from a clinical perspective. Transl. Sport Med..

[34] Hu Y.F., Chen Y.J., Lin Y.J., Chen S.A. (2015). Inflammation and the pathogenesis of atrial fibrillation. Nat. Rev. Cardiol..

[35] Datta S.D., Talwar A., Lee J.T. (2020). A Proposed Framework and Timeline of the Spectrum of Disease Due to SARS-CoV-2 Infection: Illness Beyond Acute Infection and Public Health Implications. JAMA.

[36] Milovancev A., Avakumovic J., Lakicevic N., Stajer V., Korovljev D., Todorovic N., Bianco A., Maksimovic N., Ostojic S., Drid P. (2021). Cardiorespiratory Fitness in Volleyball Athletes Following a COVID-19 Infection: A Cross-Sectional Study. Int. J. Environ. Res. Public Health.

[37] Ganesananthan S., Rajkumar C.A., Foley M., Thompson D., Nowbar A.N., Seligman H., Petraco R., Sen S., Nijjer S., Thom S.A. (2022). Cardiopulmonary exercise testing and efficacy of percutaneous coronary intervention: A substudy of the ORBITA trial. Eur. Heart J..

[38] Jimeno-Almazán A., Pallarés J.G., Buendía-Romero Á., Martínez-Cava A., Franco-López F., Sánchez-Alcaraz Martínez B.J., Bernal-Morel E., Courel-Ibáñez J. (2021). Post-COVID-19 Syndrome and the Potential Benefits of Exercise. Int. J. Environ. Res. Public Health.

[39] Faghy M.A., Ashton R.E.M., Parizher G., Smith A., Arena R., Gough L.A., Emery M.S. (2022). COVID-19 and elite sport: Cardiovascular implications and return-to-play. Prog. Cardiovasc. Dis..

[40] Malhotra R., Bakken K., D’Elia E., Lewis G.D. (2016). Cardiopulmonary Exercise Testing in Heart Failure. JACC Heart Fail..

[41] Samper-Pardo M., León-Herrera S., Oliván-Blázquez B., Gascón-Santos S., Sánchez-Recio R. (2023). Clinical characterization and factors associated with quality of life in Long COVID patients: Secondary data analysis from a randomized clinical trial. PLoS ONE.

[42] Hausswirth C., Schmit C., Rougier Y., Coste A. (2023). Positive Impacts of a Four-Week Neuro-Meditation Program on Cognitive Function in Post-Acute Sequelae of COVID-19 Patients: A Randomized Controlled Trial. Int. J. Environ. Res. Public Health.

